# Divergent Effects of Arsenic on NF-κB Signaling in Different Cells or Tissues: A Systematic Review and Meta-Analysis

**DOI:** 10.3390/ijerph13020163

**Published:** 2016-01-26

**Authors:** Meng Wei, Jiaming Liu, Mengchuan Xu, Dongsheng Rui, Shangzhi Xu, Gangling Feng, Yusong Ding, Shugang Li, Shuxia Guo

**Affiliations:** Department of Public Health, Shihezi University School of Medicine, Shihezi 832000, Xinjiang, China; m15299953350@163.com (M.W.); liujiaming@shzu.edu.cn (J.L.); 18097533626@163.com (M.X.); ruidongsheng@gmail.com (D.R.); shexxushangzhi@sohu.com (S.X.); 18139260682@189.cn (G.F.); tianmajuechen@alijun.com (Y.D.)

**Keywords:** arsenic, NF-κB, meta-analysis

## Abstract

Arsenic is ubiquitously present in human lives, including in the environment and organisms, and has divergent effects between different cells and tissues and between different exposure times and doses. These observed effects have been attributed to the nuclear transcription factor kappa B(NF-κB) signaling pathway. Herein, a meta-analysis was performed by independently searching databases including the Cochrane Library, PubMed, Springer, Embase, and China National Knowledge Infrastructure, to analyze effects of arsenic exposure on NF-κB signaling. Compared to controls, in the exposed group, p-IκB levels were found to be 8.13-fold higher (95% CI, 2.40–13.85; *Z* = 2.78; *p* = 0.005), IκB levels were 16.19-fold lower (95% CI, −27.44–−4.94; Z = 2.78; *p* = 0.005), and NF-κBp65 levels were 0.77-fold higher (95% CI, 0.13–1.42; *Z* = 2.34; *p* = 0.02) for normal cells and tissue, while NF-κBp65 levels were 4.90-fold lower (95% CI, −8.49–1.31; *Z* = 2.62; *p* = 0.009), NF-κB activity was 2.45-fold lower (95% CI, −3.66–1.25; *Z* = 4.00; *p* < 0.0001), and DNA-binding activity of NF-κB was 9.75-fold lower (95% CI, −18.66–4.54; *Z* = 2.15; *p* = 0.03) for abnormal cells and tissue. Short exposure to high arsenic doses activated the NF-κB signaling pathway, while long exposure to low arsenic doses suppressed NF-κB signaling pathway activation. These findings may provide a theoretical basis for injurious and therapeutic mechanisms of divergent effects of arsenic.

## 1. Introduction

Arsenic exists ubiquitously on earth including in the earth’s crust, in soil, in water, in air, in food, and in organisms [1,2]. The International Agency for Research on Cancer (IARC) has classified arsenic and its compounds as Group 1 carcinogens. Nearly 200 million people worldwide are threatened by arsenic poisoning, and the number of people suffering from arsenic poisoning due to water contamination alone is more than 3 million in China [3]. Epidemic investigations and studies have shown that inorganic arsenic may increase the risk of many cancers including bladder, kidney, liver, lung, prostate, and skin cancer [4,5,6,7,8]. The risks of other diseases including cardiovascular disease [9], hypertension [10], and diabetes [11] are also increased by arsenic. Nevertheless, arsenic was recently used as an experimental anti-tumor drug in clinical treatment [12]. Investigations into arsenic poison effects and related molecular mechanisms as well as those of the therapeutic effects of arsenic have revealed that NF-κB signaling plays an important role in mediating the poison effects as well as the therapeutic effects of arsenic. It has furthermore been shown that arsenic activates NF-κB signaling in normal cells or tissues [13,14,15], while suppressing the NF-κB signaling pathway in tumor cells or inflammatory tissue [16]. The aim of this meta-analysis was to retrieve experimental data on arsenic and the NF-κB signaling pathway published both in China and worldwide in the past few years. The meta-analysis also included comprehensive analysis of this data as a means of providing a theoretical basis for the injury and treatment mechanisms of arsenic.

## 2. Materials and Methods

### 2.1. Search Strategy

Using the PICO principle [17], searches were performed using the following electronic databases: the Cochrane Library, PubMed, Embase, Springer, Web of Science, China Science and Technology Journal Database (CSTJ), and China National Knowledge Infrastructure (CNKI) (last search conducted in September 2015). The key search string was (arsenic) AND (NF-κB) OR (NF-KappaB) OR (IKK) OR (IκB) OR (Nuclear transcription factor kappa B) OR (Inhibitor of kappa B kinase) OR (Inhibitor of kappa B).

### 2.2. Eligibility Criteria

The eligibility criteria for including articles in the meta-analysis were as follows: any animal and cells lines, gender not limited, published in either Chinese or English. Arsenic model groups induced by any kind of arsenic, and its compounds were used as the experimental groups, and the untreated served as control groups. If various doses of arsenic were used in a study, the highest dose was chosen for this analysis. If various exposure times of arsenic were used in a study, the longest time was chosen for this analysis.

### 2.3. Exclusion Criteria

The exclusion criteria were as follows: (1) repeat publications; (2) incomplete information; (3) insufficient or insignificant statistical data; (4) unrelated to the study objectives; (5) lack of appropriate controls; and (6) review articles.

### 2.4. Outcome Indicators

Nuclear transcription factor kappa B(NF-κB), inhibitor of kappa B(IκB), phosphorylase inhibitor of kappa B(p-IκB), inhibitor of kappa B kinase (IKK), nuclear transcription factor kappa B activity (NF-κB activity), DNA-binding activity of the nuclear transcription factor kappa B (DNA-binding activity of NF-κB), nuclear transcription factor kappa B p65 (NF-κBp65), and nuclear transcription factor kappa B mRNA(NF-κB mRNA) were included as outcome indicators.

### 2.5. Data Extraction

Two reviewers (Shugang Li and Meng Wei) independently screened full-length articles. The following information was extracted from the complete manuscripts of each qualified study: publication characteristics (title of the study, first author, publication date, and journal/magazine title), baseline data (n, mean ± standard deviation [SD]) for the experimental and control groups, subject characteristics (source of cells and tissue, arsenic doses and exposure times), outcome indicators, and the source of indicator estimates. When the two reviewers’ opinions differed, Shuxia Guo, who teaches meta-analysis at Shihezi University School of Medicine, was asked to make the final decision regarding the results.

### 2.6. Data Analysis

Twenty-seven articles were analyzed in Review Manager Version 5.2 (The Nordic Cochrane Centre, The Cochrane Collaboration, 2012, Portland Oregon, OR, USA) and Stata 12.0 (StataCorp., College Station, Texas, TX, USA). Significant heterogeneity was detected (*p* < 0.05, *I*^2^ > 75%) and a random effects model was therefore applied for the meta-analysis. A multivariate meta-regression analysis was performed to determine the source of heterogeneity and continuous variables were estimated as standardized mean differences (SMDs) with 95% confidence intervals (CIs) between the arsenic treated groups and control groups. All reported *p*-values are two-sided and a significance level of 0.05 was used. For additional insight, subgroup analyses were performed based on exposure dose (≤5 μmol/L or ˃5 μmol/L *in vivo*; ≤5 mg/kg or ˃5 mg/kg *in vitro*) and exposure time (≤24 h or ˃24 h), based on the median of the indexes reported in the papers, to determine the factors associated with differences in the outcome indicators across different studies. Small-study effects were explored using funnel plots and Egger’s tests and studysensitivities were assessed.

## 3. Results

### 3.1. Study Characteristics

**Figure 1:**
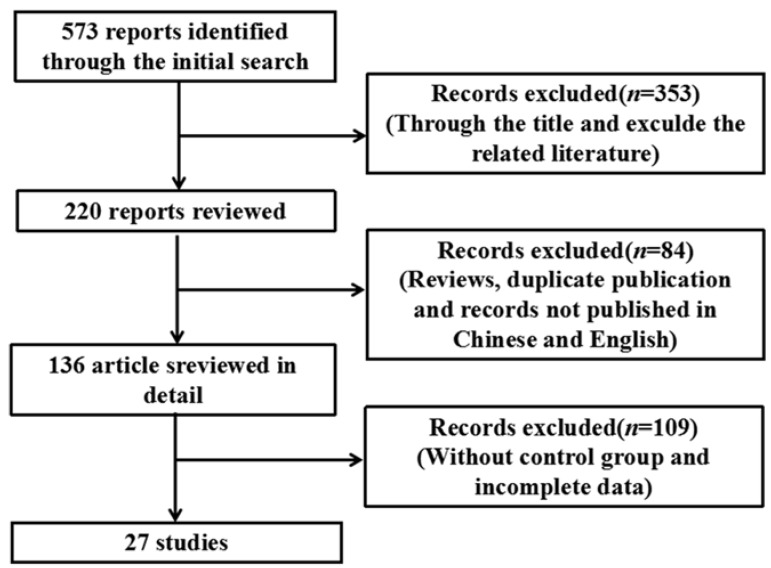
Flowchart detailing the publication search strategy.

Using the search strategy described in Section 2, 573 relevant articles were identified (Figure 1), of which 27 were used for the meta-analysis based on the eligibility and exclusion criteria (Table 1). Various cell lines and animals were used as arsenic model groups in these studies, and in each study, the effect of arsenic on the NF-κB signaling pathway was assessed. The arsenic model groups were primarily cell lines and animals treated with arsenicin various forms (e.g., arsenite, As_2_O_3_, and dimethylarsinic acid), and the control models were blankcontrols. Arsenic exposure time varied among the studies and was categorized as ≤24 h (*n* = 15) or >24 h (*n* = 12). Arsenic dose also varied among the studies, and was categorized as either low dose (*n* = 16) or high dose (*n* = 11). NF-κB signaling pathway indices (*i.e.*, NF-κB, IκB, p-IκB, NF-κB mRNA, NF-κB activity, IKK, DNA-binding activity of NF-κB, and NF-κBp65) were examined either in normal cells or tissues (*n* = 9) or in inflammatory and cancer cells or tissue (*n* = 18).

**Table 1:** Characteristics of the studies included in the meta-analysis.

First Author (Year)	Language	*n*	Type of Cells or Tissue	Type of Arsenical Compounds	Period of Arsenic	Dose of Arsenic	Outcome Indicators
Jyotirmoy Ghosh 2009 [13]	English	6	Normal cells	NaAsO_2_	≤24 h	≤5 μmol/L	1,8
Shi-Yi Liu 2014 [14]	Chinese	3	Normal cells	NaAsO_2_	˃24 h	≤5 μmol/L	2
Daniella M. B. Kerbauy 2005 [15]	English	3	Cancer cells	As_2_O_3_	≤24 h	>5 μmol/L	2,8
Yi Hao 2012 [16]	Chinese	3	Cancer cells	As_2_O_3_	≤24 h	>5 μmol/L	1,3,5,6
François Binet 2010 [17]	English	3	Normal cells	As_2_O_3_	≤24 h	≤5 μmol/L	7
Kumar Felix 2005 [18]	English	3	Normal cells	NaAsO_2_	≤24 h	>5 μmol/L	3,7,8
Xue-Zhong Gong 2015 [19]	English	3	Normal cells	NaAsO_2_	≤24 h	≤5 μmol/L	8
Si-Qi Cao 2015 [20]	Chinese	20	Normal cells	DMAѴ	˃24 h	>5 mg/kg	1,4,5
Xiao-Yan Qu 2012 [21]	English	3	Cancer cells	As_2_O_3_	˃24 h	≤5 μmol/L	1
Yu Hu 2002 [22]	English	3	Normal cells	NaAsO_2_	≤24 h	≤5 μmol/L	7
Lin-Fu Zhou 2006 [23]	English	6	Inflammatory tissue	As_2_O_3_	≤24 h	≤5 mg/kg	3,6
Hye Ran Lee 2008 [24]	English	3	Cancer cells	As_2_O_3_	≤24 h	>5 μmol/L	2,3,8
Min Jeong Kim 2014 [25]	English	3	Cancer cells	As_4_O_6_	≤24 h	≤5 μmol/L	2,3,5
De-Lin Wang 2007 [26]	Chinese	15	Cancer cells	As_2_O_3_	˃24 h	≤5 μmol/L	1,3,4,5,7
Stephan Mathas 2003 [27]	English	3	Cancer cells	NaAsO_2_	˃24 h	>5 μmol/L	7
Jing Qiu 2008 [28]	Chinese	9	Cancer cells	As_2_O_3_	˃24 h	≤5 mg/kg	1,3,8
Ruben Ruiz-Ramos 2009 [29]	English	3	Cancer cells	NaAsO_2_	≤24 h	>5 μmol/L	2,8
Yong-Shen Fan 2008 [30]	Chinese	5	Cancer cells	As_2_O_3_	˃24 h	≤5 μmol/L	6
Shu-jian Wang 2008 [31]	Chinese	3	Cancer cells	As_2_O_3_	˃24 h	≤5 μmol/L	4
Yi-Fang Mei 2006 [32]	Chinese	4	Inflammatory cells	As_2_O_3_	˃24 h	>5 μmol/L	8
Robert R. Roussel 2000 [33]	English	3	Normal cells	NaAsO_2_	≤24 h	≤5 μmol/L	2,3,6,8
Ke-Xin Zhang 2015 [34]	English	6	Normal cells	As_2_O_3_	˃24 h	>5 μmol/L	4
Xiao-Wei Xu 2005 [35]	Chinese	3	Cancer cells	As_2_O_3_	≤24 h	≤5 μmol/L	8
Xiao-Hong Zhang 2004 [36]	Chinese	3	Cancer cells	As_2_O_3_	≤24 h	≤5 μmol/L	3,5
Yao Zhang 2008 [37]	Chinese	3	Cancer cells	As_2_O_3_	≤24 h	>5 μmol/L	5
P. B. Tchounwou 2002 [38]	English	3	Cancer cells	As_2_O_3_	˃24 h	≤5 μg/mL	8
Yi-Fang Mei 2011 [39]	English	3	Inflammatory cells	As_2_O_3_	˃24 h	≤5 μmol/L	7

**Note:** *n* = number of experimental cells or animals group; 1 = IKK, 2 = p-IκB, 3 = IκB, 4 = NF-κB mRNA, 5 = NF-κBp65, 6 = NF-κBactivity, 7 = DNA-binding activity of NF-κB and 8 = NF-κB.

### 3.2. Meta-Analysis of Arsenic Exposure Effects on the NF-κB Signaling Pathway

#### 3.2.1. Effects of Arsenic Exposure on p-IκB

**Figure 2:**
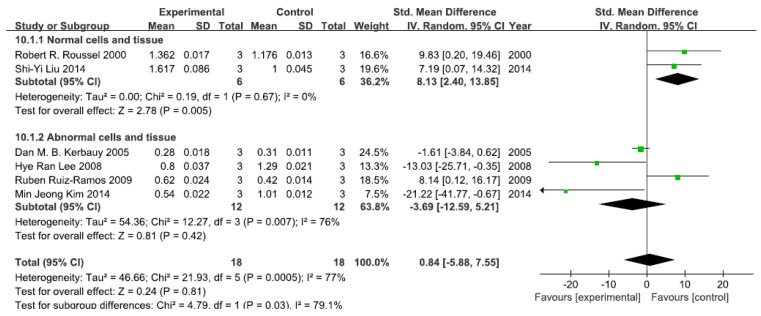
Effects of arsenic on phosphorylase inhibitor of kappa B (p-IκB). Forest plot showing the impact of arsenic treatment on p-IκB compared with controls. Abbreviations: SMD = standardized mean difference, IV = independent variable, 95% CI = 95% confidence interval.

A total of six studies assessed p-IκB levels. A pooled analysis showed that p-IκB levels were 8.13-fold higher in the exposed group than in the control group (95% CI, 2.40–13.85; *Z* = 2.78; *p* = 0.005) for normal cells and tissue, with no significant heterogeneity (*p* = 0.65; *I*^2^ = 0%; Figure 2). Pooled analysis furthermore showed that p-IκB levels were 3.69-fold lower in the exposed group than in the control group (95% CI, −12.59–5.21; *Z* = 0.81; *p* = 0.42) for abnormal cells and tissue, with significant heterogeneity (*p* = 0.007; *I*^2^ = 76%; Figure 2).

#### 3.2.2. Effects of Arsenic Exposure on IκB

**Figure 3:**
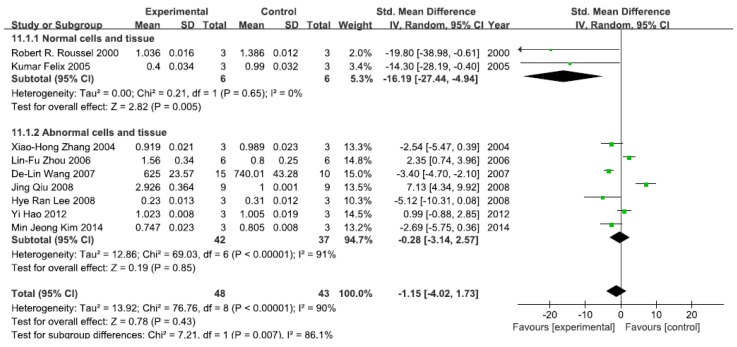
Effects of arsenic on inhibitor of kappa B(IκB). Forest plot showing the impact of arsenic treatment on IκB compared with controls. Abbreviations: SMD = standardized mean difference, IV = independent variable, 95% CI = 95% confidence interval.

A total of nine studies assessed IκB levels. A pooled analysis showed that IκB levels were 16.19-fold lower in the exposed group than in the control group (95% CI, −27.44–−4.94; Z = 2.78; *p* = 0.005) for normal cells and tissue, with no significant heterogeneity (*p* = 0.65; *I*^2^ = 0%; Figure 3). Pooled analysis furthermore showed that IκB levels were 0.28-fold lower in the exposed group than in the control group (95% CI, −3.14–2.57; Z = 0.19; *p* = 0.85) for abnormal cells and tissue, with significant heterogeneity (*p* < 0.0001; *I*^2^ = 91%; Figure 3).

#### 3.2.3. Effects of Arsenic Exposure on NF-κBp65

**Figure 4:**
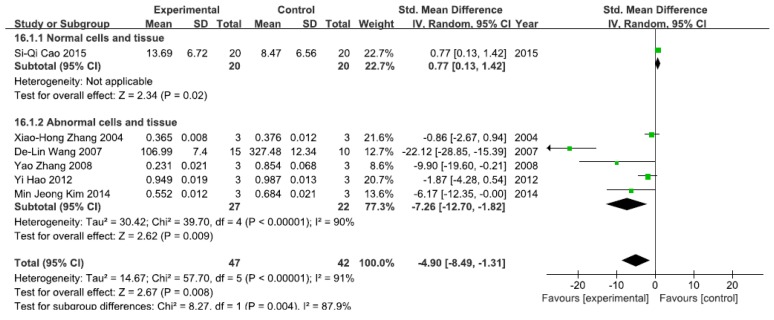
Effects of arsenic on nuclear transcription factor kappa B p65(NF-κBp65). Forest plot showing the impact of arsenic treatment on NF-κBp65 compared with controls. Abbreviations: SMD = standardized mean difference, IV = independent variable, 95% CI = 95% confidence interval.

A total of six studies assessed NF-κBp65 levels. A pooled analysis showed that NF-κBp65 levels were 0.77-fold higher in the exposed group than in the control group (95% CI, 0.13–1.42; *Z* = 2.34; *p* = 0.02) for normal cells and tissue (Figure 4), while pooled analysis showed that NF-κBp65 levels were 4.90-fold lower in the exposed group than in the control group (95% CI, −8.49–1.31; *Z* = 2.62; *p* = 0.009) for abnormal cells and tissue, with significant heterogeneity (*p* < 0.0001; *I*^2^ = 90%; Figure 4).

#### 3.2.4. Effects of Arsenic Exposure on NF-κB

**Figure 5:**
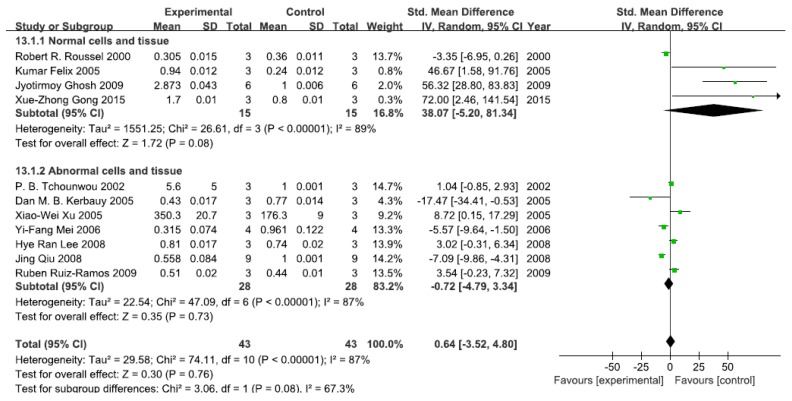
Effects of arsenic on nuclear transcription factor kappa B(NF-κB). Forest plot showing the impact of arsenic treatment on NF-κB compared with controls. Abbreviations: SMD = standardized mean difference, IV = independent variable, 95% CI = 95% confidence interval.

A total of 11 studies assessed NF-κB levels. A pooled analysis showed that NF-κB levels were 38.07-fold higher in the exposed group than in the control group (95% CI, −5.20–81.34); *Z* = 1.72; *p* = 0.08) for normal cells and tissue, with significant heterogeneity (*p* < 0.0001; *I*^2^ = 89%; Figure 5). Pooled analysis furthermore showed that NF-κB levels were 0.72-fold lower in the exposed group than in the control group (95% CI, −4.79–3.34; *Z* = 0.35; *p* = 0.73) for abnormal cells and tissue, with significant heterogeneity (*p* < 0.0001; *I*^2^ = 87%; Figure 5).

#### 3.2.5. Effects of Arsenic Exposure on IKK

**Figure 6:**
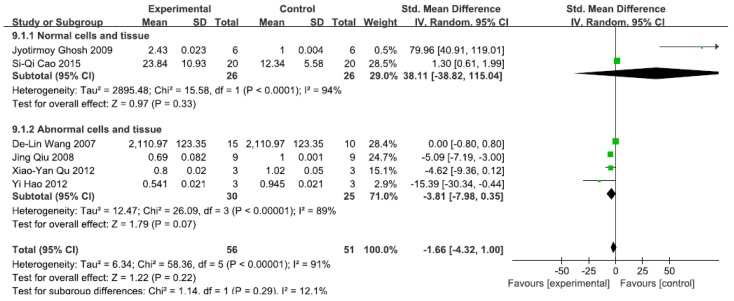
Effects of arsenic on inhibitor of kappa B kinase(IKK). Forest plot showing the impact of arsenic treatment on IKK compared with controls. Abbreviations: SMD = standardized mean difference, IV = independent variable, 95% CI = 95% confidence interval.

A total of 11 studies assessed IKK levels. A pooled analysis showed that IKK levels were 38.11-fold higher in the exposed group than in the control group (95% CI, −38.82–115.04; *Z* = 0.97; *p* = 0.33) for normal cells and tissue, with significant heterogeneity (*p* < 0.0001; *I*^2^ = 94%; Figure 6). Pooled analysis furthermore showed that IKK levels were 3.81-fold lower in the exposed group than in the control group (95% CI, −7.98–0.35; *Z* = 1.79; *p* = 0.07) for abnormal cells and tissue, with significant heterogeneity (*p* < 0.0001; *I*^2^ = 91%; Figure 6).

#### 3.2.6. Effects of Arsenic Exposure on NF-κB Activity

**Figure 7:**
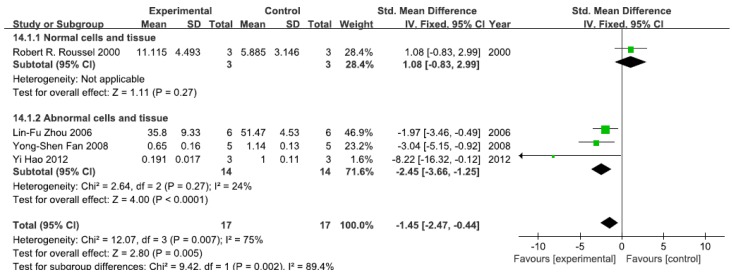
Effects of arsenic on nuclear transcription factor kappa B activity(NF-κB activity). Forest plot showing the impact of arsenic treatment on NF-κB activity compared with controls. Abbreviations: SMD = standardized mean difference, IV = independent variable, 95% CI = 95% confidence interval.

A total of four studies assessed NF-κB activity. A pooled analysis showed that NF-κB activity was 1.08-fold higher in the exposed group than in the control group (95% CI, −0.83–2.99; *Z* = 1.11; *p* = 0.27) for normal cells and tissue (Figure 7). Pooled analysis furthermore showed that NF-κB activity was 2.45-fold lower in the exposed group than in the control group (95% CI, −3.66–1.25; *Z* = 4.00; *p* < 0.0001) for abnormal cells and tissue, with no significant heterogeneity (*p* = 0.27; *I*^2^ = 24%; Figure 7).

#### 3.2.7. Effects of Arsenic Exposure on DNA-Binding Activity of NF-κB

**Figure 8:**
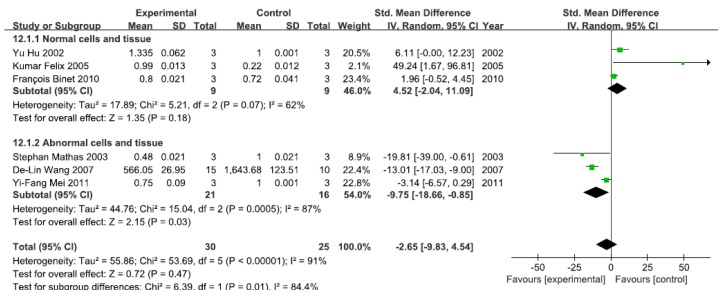
Effects of arsenic on the DNA-binding activity of the nuclear transcription factor kappa B(DNA-binding activity of NF-κB). Forest plot showing the impact of arsenic treatment on the DNA-binding activity of NF-κB compared with controls. Abbreviations: SMD = standardized mean difference, IV = independent variable, 95% CI = 95% confidence interval.

A total of six studies assessed the DNA-binding activity of NF-κB. A pooled analysis showed that the DNA-binding activity of NF-κB was 4.52-fold higher in the exposed group than in the control group (95% CI, −2.04–11.09; *Z* = 1.35; *p* = 0.18) for normal cells and tissue, with no significant heterogeneity (*p* = 0.07; *I*^2^ = 62%; Figure 8). Pooled analysis furthermore showed that the DNA-binding activity of NF-κB was 9.75-fold lower in the exposed group than in the control group (95% CI, −18.66–4.54; *Z* = 2.15; *p* = 0.03) for abnormal cells and tissue, with significant heterogeneity (*p* = 0.0005; *I*^2^ = 87%; Figure 8).

#### 3.2.8. Effects of Arsenic Exposure on NF-κB mRNA

**Figure 9:**
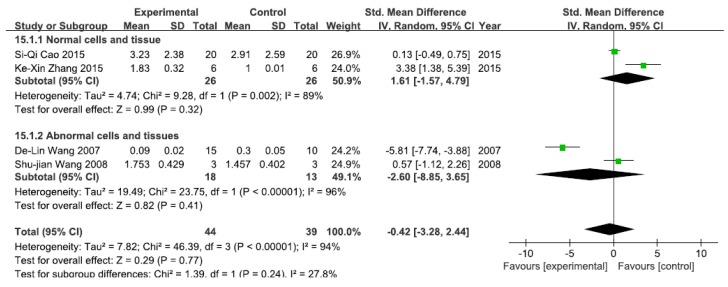
Effects of arsenic on nuclear transcription factor kappa B mRNA(NF-κB mRNA). Forest plot showing the impact of arsenic treatment on NF-κB mRNA compared with controls. Abbreviations: SMD = standardized mean difference, IV = independent variable, 95% CI = 95% confidence interval.

A total of four studies assessed NF-κB mRNA levels. A pooled analysis showed that NF-κB mRNA levels were 1.61-fold higher in the exposed group than in the control group (95% CI, −1.57–4.79; Z = 0.99; *p* = 0.32) for normal cells and tissue, with significant heterogeneity (*p* = 0.002; *I*^2^ = 89%; Figure 9). Pooled analysis furthermore showed that NF-κB mRNA levels were 2.60-fold lower in the exposed group than in the control group (95% CI, −8.85–3.65; *Z* = 2.15; *p* = 0.41) for abnormal cells and tissue, with significant heterogeneity (*p* < 0.0001; *I*^2^ = 96%; Figure 9).

### 3.3. Subgroup Analyses of the Effects of Arsenic Exposure

**Figure 10:**
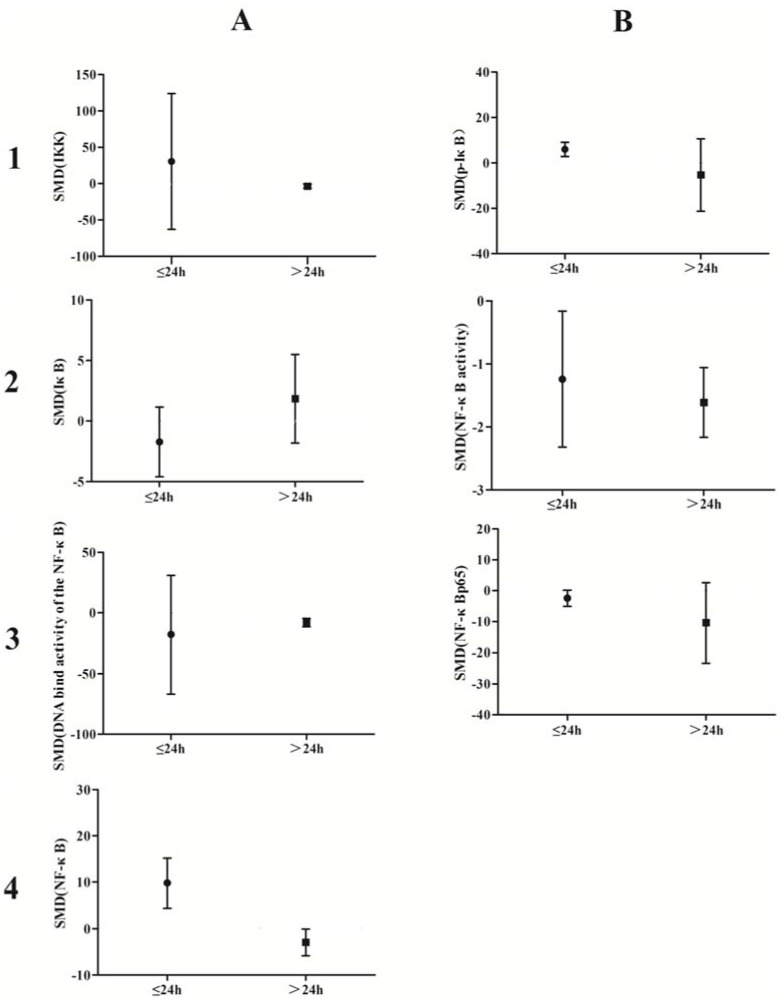
Subgroup analyses to determine the effects of arsenic on NF-κB. Based on exposure timeanalysis, arsenic exposure times of ≤24 h promote phosphorylation of IκB(B1), induce weak NF-κB activity(B2), and increase NF-κBp65 expression (B3), while arsenic exposure times ˃24 h suppresses NF-κB activity(B2) and attenuates the DNA-binding activity of NF-κB(A3). Abbreviations: SMD = standardized mean difference. **1**, **2**, **3**, and **4** represent the row number, **A** and **B** refer to the column number.

**Figure 11:**
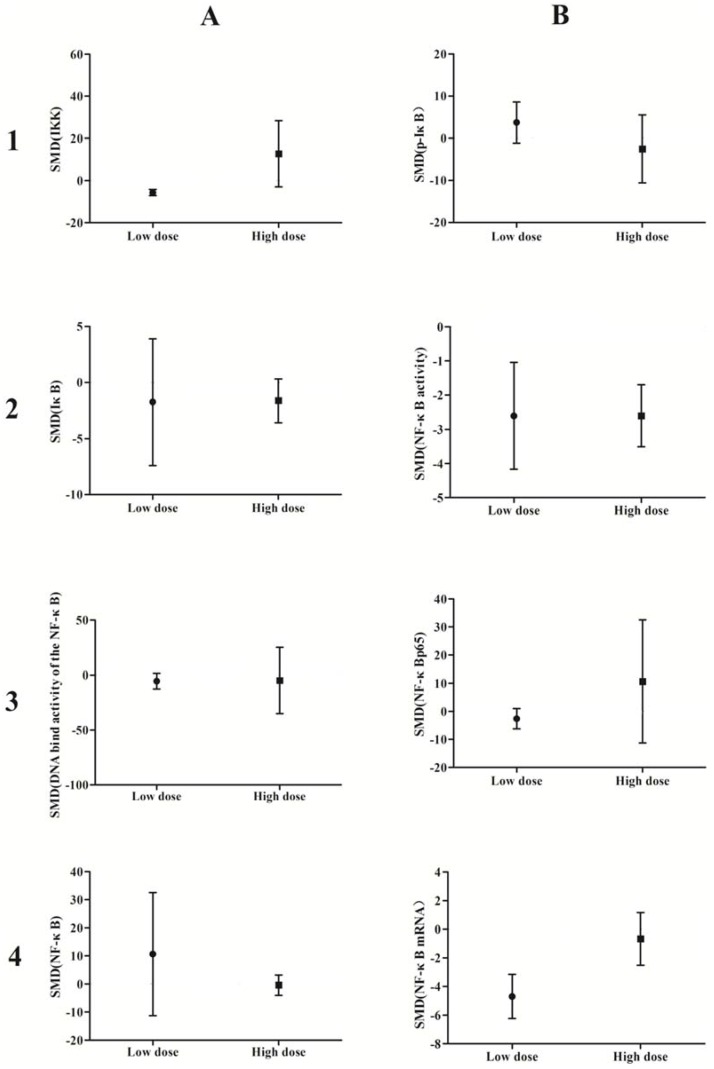
Subgroup analyses to determine the effects of arsenic on NF-κB. Based on exposure dose analysis, low doses of arsenic exposure were found to reduce IKK(A1), NF-κB activity (B2), and NF-κBp65 expression. Abbreviations: SMD = standardized mean difference. **1**, **2**, **3**, and **4** represent the row number, **A** and **B** refer to the column number.

A subgroup analysis based on exposure dose (≤5 μmol/L *vs.* ˃5 μmol/L *in vivo*, ≤5 mg/kg *vs.* ˃5 mg/kg *in vitro*) and time (≤24 h *vs.* ˃24 h) was conducted. The analysis demonstrated that arsenic exposure times of ≤24 h promote phosphorylation of IκB (*p* = 0.0002), induce weak NF-κB activity (*p* = 0.02), and increase NF-κBp65 expression (*p* = 0.04), while arsenic exposure times ˃24 h suppresses NF-κB activity (*p* = 0.001) and attenuates the DNA-binding activity of NF-κB (*p* = 0.007) (Figure 10). Low doses of arsenic exposure were found to reduce IKK (*p* < 0.00001), NF-κB activity (*p* = 0.001), and NF-κBp65 expression (Figure 11).

### 3.4. Small-Study Effect Evaluation

**Figure 12:**
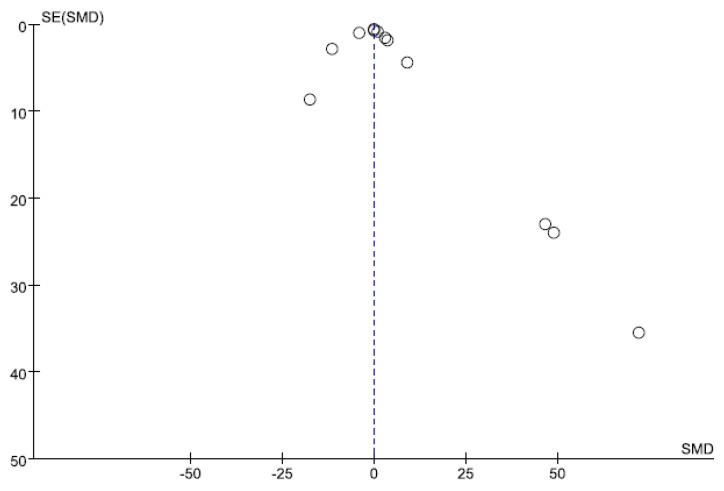
Funnel plot for NF-κB. The blue-dotted line shows the overall estimated standard mean difference. Evidence for publication bias was not found (*p* = 0.441). Abbreviations: SMD = standard mean difference, SE = standard error.

Visual inspection of the funnel plot and Egger’s test results showed no evidence of significant small-study effects (*p* = 0.441; Figure 12).

### 3.5. Sensitivity Analysis

**Figure 13:**
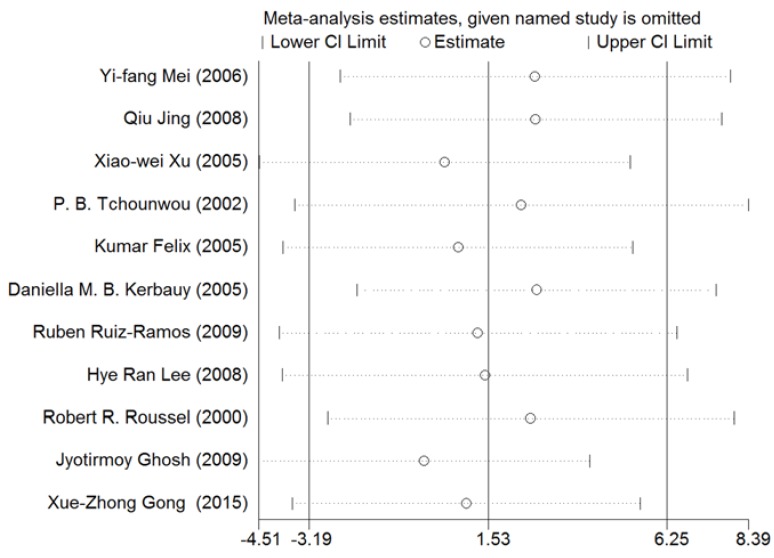
Sensitivity analysis for NF-κB. Stable results were observed for all studies, indicating that no individual study influenced the combined results. Abbreviations: CI = confidence interval.

A sensitivity analysis was conducted for NF-κB. As shown in Figure 13, the results for all studies were stabilized, and thus, no individual study was found to influence the combined results.

### 3.6. Meta-Regression Analysis of Arsenic Exposure Effects

A multivariate meta-regression analysis of the retrieved data revealed that the effects of arsenic exposure time (*p* = 0.013) and dose (*p* = 0.022) were significantly associated with differences in NF-κB levels.

## 4. Discussion

The findings of this meta-analysis reveal that arsenic activates the NF-κB signaling pathway in normal cells or tissues; however, the antitumor and anti-inflammatory effects of arsenic are accompanied by partial suppression of the NF-κB signaling pathway. Short arsenic exposure may promote the activation of NF-κB signaling, while long exposure may suppress the NF-κB signaling pathway. Compared with high doses, low doses of arsenic were shown to weaken NF-κB signaling pathway expression. These findings provide a theoretical foundation for understanding the contrasting toxic and therapeutic effects of arsenic.

**Figure 14:**
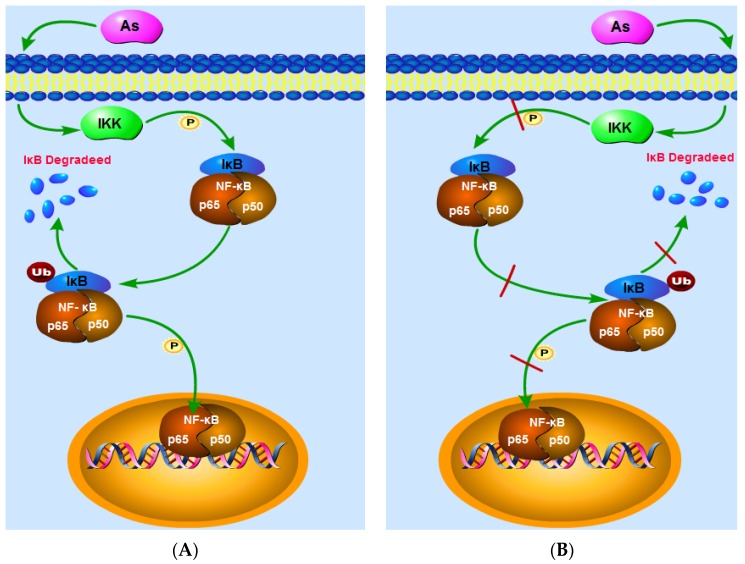
The NF-κB signaling pathway. (**A**) shows that at high doses, the action of IKK enhances IκB phosphorylation, degrades IκB, reduces the content of IκB in the cytoplasm, and increases the DNA-binding activity of NF-κB in normal tissues or cells, thereby enhancing the expression of NF-κBp65 and NF-κB; (**B**) indicates that, in inflammatory or tumor cells, arsenic reduces cytoplasmic IKK levels, suppresses IκB phosphorylation, attenuates IκB degradation, increases cytoplasmic IκB levels, and weakens the DNA-binding activity of NF-κB, thereby decreasing the expression levels of NF-κB and NF-κBp65.

Arsenic has been reported to damage the human body via activation of the NF-κB signaling pathway [18,19,20]. Here, we show that at high doses, the action of IKK enhances IκB phosphorylation, degrades IκB, reduces the content of IκB in the cytoplasm, and increases the DNA-binding activity of NF-κB in normal tissues or cells, thereby enhancing the expression of NF-κBp65 and NF-κB (Figure 14A). Arsenic may therefore increase cytoplasmic IKK levels, thereby enhancing IKK phosphorylation, which, in turn, results in an increase in IKK activity, resulting in IκB phosphorylation and subsequent cytoplasmic degradation of IκB [13,14]. Elsewhere, arsenic has been shown to boost the DNA-binding activity of NF-κB and to enhance NF-κB mRNA expression, thereby promoting the expression of NF-κB [21]. These effects may result from arsenic-induced increases in cellular reactive oxygen species, which, in turn, promote NF-κB expression. Arsenic can also decrease NF-κB levels in the cytoplasm and increase the nuclear transcription of NF-κB [22]. It was recently also shown that arsenic has a therapeutic effect on tumors and inflammation, and one of the main mechanisms by which arsenic exerts such effects is by inhibiting the activation of NF-κB signaling [23,24,25,26].

Arsenic has been shown to inhibit the activation of NF-κB signaling in tumors and inflammatory cells [27,28,29,30,31]. The findings of our analysis indicate that, in inflammatory or tumor cells, arsenic reduces cytoplasmic IKK levels, suppresses IκB phosphorylation, attenuates IκB degradation, increases cytoplasmic IκB levels, and weakens the DNA-binding activity of NF-κB, thereby decreasing the expression levels of NF-κB and NF-κBp65 (Figure 14B). On the one hand, the inhibition of IKK activity in the presence of arsenic may be a result of arsenic acting on and affecting the lysosome, thereby diminishing IKK activity [32], reducing IκB phosphorylation, and inhibiting the degradation of IκB [33]. On the other hand, arsenic may act by reducing the NF-κB connected with DNA activity and inhibiting the translocation of NF-κB into the nucleus, thereby suppressing NF-κB mRNA and then protein expression [34,35,36]. Some researchers believe that arsenic exposure leads to elevatedreactive oxygen species levels which inhibit NF-κB activity, resulting in decreased expression of NF-κB [37,38,39].

Different arsenic concentrations and exposure times may account for the different effects of arsenic on NF-κB activity in the same cells. Huand colleagues [22] found that short (3–24 h) periods of As_2_O_3_ administration (<25 μmol/L) in GM847 cells activates NF-κB signaling, while prolonged exposure (10 weeks) to arsenic (0.1~0.5 μmol/L) inhibits NF-κB expression in these same cells. Liao and colleagues [40] showed that 105 μmol/L doses of As_2_O_3_ for 48 h in HEK cells significantly increased NF-κB activity, while exposure to 5 μmol/L As_2_O_3_ induced significant inhibition of NF-κB activity accompanied by an increase in the apoptosis ratio. These findings are consistent with our meta-analysis results: high dose exposure and short exposure may lead to NF-κB signaling pathway activation, while low dose exposure and long exposure suppresses the activation of NF-κB signaling.

Arsenic is widely distributed in nature and may therefore have a significant impact on long-term human development. Long-term chronic arsenic exposure can affect human health, specifically by affecting the cardiovascular [41], respiratory [42], gastrointestinal [43] systems among others. In recent years, arsenic has been shown to also play important antitumor and anti-inflammatory roles in disease systems including breast cancer [44], leukemia [45], and rheumatoid arthritis [39], exhibiting curative effects in these diseases. At present, some scholars consider the NF-κB signaling pathway to be one of the important mechanisms underlying the poison effects as well as the antitumor and anti-inflammatory effects of arsenic. In the application of arsenic as a potential treatment for cancer, therefore, the dose and treatment time of choice is particularly important, considering that inappropriate dosage and exposure time may increase the extent of the damage to the body.

## 5. Conclusions

As described in Section 3 and Section 4, the present study demonstrates that arsenic may cause toxic injury via NF-κB signaling pathway activation in normal cells or tissue; however, in cancers or inflammatory cells and tissues, arsenic may have certain therapeutic effects which result from suppression of the NF-κB signaling pathway. These findings contribute to the potential development of therapeutic arsenic and provide a theoretical basis for the implementation of such therapies.
